# Life Expectancy in Duchenne Muscular Dystrophy: Reproduced Individual Patient Data Meta-analysis

**DOI:** 10.1212/WNL.0000000000012910

**Published:** 2021-12-07

**Authors:** Jonathan Broomfield, Micki Hill, Michela Guglieri, Michael Crowther, Keith Abrams

**Affiliations:** From the Department of Health Sciences (J.B., M.H.), University of Leicester; Institute of Human Genetics (M.G.), Newcastle University, UK; Department of Medical Epidemiology and Biostatistics (M.C.), Karolinska Institute, Stockholm, Sweden; and Centre for Health Economics (K.A.), University of York, UK.

## Abstract

**Background and Objectives:**

Duchenne muscular dystrophy (DMD) is a rare progressive disease that is often diagnosed in early childhood and leads to considerably reduced life expectancy; because of its rarity, research literature and patient numbers are limited. To fully characterize the natural history, it is crucial to obtain appropriate estimates of the life expectancy and mortality rates of patients with DMD.

**Methods:**

A systematic review of the published literature on mortality in DMD up to July 2020 was undertaken, specifically focusing on publications in which Kaplan-Meier (KM) survival curves with age as a timescale were presented. These were digitized, and individual patient data (IPD) were reconstructed. The pooled IPD were analyzed with the KM estimator and parametric survival analysis models. Estimates were also stratified by birth cohort.

**Results:**

Of 1,177 articles identified, 14 publications met the inclusion criteria and provided data on 2,283 patients, of whom 1,049 had died. Median life expectancy was 22.0 years (95% confidence interval [CI] 21.2, 22.4). Analyses stratified by 3 time periods in which patients were born showed markedly increased life expectancy in more recent patient populations; patients born after 1990 have a median life expectancy of 28.1 years (95% CI 25.1, 30.3).

**Discussion:**

This article presents a full overview of mortality across the lifetime of a patient with DMD and highlights recent improvements in survival. In the absence of large-scale prospective cohort studies or trials reporting mortality data for patients with DMD, extraction of IPD from the literature provides a viable alternative to estimating life expectancy for this patient population.

Duchenne muscular dystrophy (DMD) is an X-linked, muscle degeneration disease affecting males nearly exclusively. It is a rare disease with a global prevalence of 1 in 3,500 to 5,000 male births.^1^ Although corticosteroids are the mainstay treatment, there is currently no cure. To be reimbursed by health agencies, companies that develop new treatments need to show cost-effectiveness, which requires accurate modeling of the natural history of the disease, including mortality.

DMD mortality has been published in isolation across different countries, sources, and time periods, typically representing the experience of a single practice or selective population. Previous estimates of life expectancy have been reported in the literature as 25 years,^2,3^ while in more recent years, this has increased to 31.7 (95% confidence interval [CI] 27.4, 36.0).^4^ Many studies report trends of increasing life expectancy with time.^1,5-7^

A systematic review was recently conducted and obtained a single estimate of life expectancy of 29.9 years (95% CI 26.5, 30.8) in ventilated patients with DMD.^8^ While the review provides an excellent summary of the published literature, survival across the whole disease pathway, rather than just the median, is needed to appropriately characterize a natural history model.

This article therefore aims to extend beyond these single summary estimates of life expectancy and provide comprehensive survival probabilities/mortality rates at different ages. This was achieved by performing a systematic review of the published literature on DMD life expectancy, reconstructing individual patient data (IPD) and calculating pooled estimates. These can be used in future natural history or economic modeling of DMD.

## Methods

### Systematic Review

A systematic review was performed on PubMed on July 31, 2020, and publications on DMD mortality before this date were identified. The following search terms were used: (1) Duchenne muscular dystrophy or DMD; (2) survival or mortality or death or life expectancy; and (3) 1 and 2.

The citations of a subset of the results, which represented systematic reviews and meta-analyses, were also reviewed for inclusion. The additional search terms included for this review were (4) systematic review or meta-analysis and (5) 3 and 4.

Searches 3 and 5 were used to conduct the review. There were no exclusions based on region, language, or time. Only full texts that were freely available to the Universities of Leicester and Sheffield were included in the review. The publications were required to report at least 1 Kaplan-Meier (KM) curve for survival in patients with DMD, which was generally confirmed by articles reporting genetic diagnosis. The KM curve had to be calculated as all-cause survival, with age as a timescale. Last, the reported KM curve had to be able to be digitized, requiring the number of patients at risk to be reported and the curve to be of suitable digital quality. When KM curves were stratified by a covariate, the number of patients in each stratum had to be reported because these curves must be digitized separately.

The review was restricted to all-cause mortality to ensure that the outcome was comparable across studies, and it was assumed that most deaths in these patients would be related to DMD. Age was chosen as the timescale because this provided clinically meaningful survival estimates and, for example, did not require knowledge of age at diagnosis.

If multiple KM curves were presented in a single article relating to the same DMD population, all were digitized for comparison, and a joint decision was made by the authors as to which data to include in the analysis, prioritizing curves including the highest number of patients and curves of higher digital quality.

The review was carried out according to Preferred Reporting Items for Systematic Reviews and Meta-Analyses guidelines for conducting a systematic review.^9^ Two authors conducted the review separately and discussed any discrepancies. The quality of the articles included was assessed with the Joanna Briggs Institute (JBI) Critical Appraisal tool for case series because the population consists exclusively of patients with DMD^10^; if studies were assessed by the tool as being at risk of bias, the methods were repeated with these studies excluded as a sensitivity analysis.

### IPD Extraction

The KM curves for each strata were digitized with WebPlotDigitizer.^11^ The IPD were reconstructed using the approach of Guyot et al.,^12^ which was developed and implemented with the ipdfc command^13^ in Stata. Many studies reported KM curves split by levels of a discrete covariate. In these instances, each curve was digitized separately and the data from the curves were pooled. Digitization of curves was also conducted by 2 authors, with summary statistics (medians or earlier quantiles if not reached and survival probabilities) and reproduced graphs being compared to the original study graph to ensure a suitable degree of accuracy. If the digitized number of patients or deaths was greater or less than the original number by 10% for both authors, the study was deemed insufficiently digitized and excluded from analysis.

### Pooled Data Analysis

Two approaches to the data analysis were adopted: a nonparametric overview of DMD mortality and a parametric calculation of mortality rates. The data were analyzed first as a combined cohort and second by stratifying the pooled IPD into 3 cohorts based on the period of birth for each study: before 1970, 1970 to 1990, and after 1990. If period of birth for each group of patients was not explicitly reported, then it was approximated as the average age at recruitment subtracted from the midpoint of the period of recruitment. The birth cohorts were defined to evenly split patients and to investigate trends in life expectancy over time. The most recent birth cohort is likely to contain only steroid-using patients because steroid use has been the mainstay of treatment since the 1990s. The cohort analysis is also likely to represent a comparison of ventilated and nonventilated patients because ventilation was introduced in many clinical settings only in the 1990s.^8^

A nonparametric KM curve was used to summarize the survival estimates of the pooled sources. Median survival was compared in the pooled dataset and across birth cohorts.

A parametric survival model with a piecewise-constant hazard function was estimated with cut points first every 5 years and then every year from age 0 to the maximum observed death. This covered follow-up from possible diagnosis at birth to a time horizon of the oldest observed death in the dataset. This model assumes that the rate of death of patients is the same for all ages within a time period while allowing rates of death to differ across time periods. Because the rate of death in young patients is likely to be very different from the rate of death in older patients, this model choice is preferred to, for example, an exponential model that assumes the same rate of death across all ages. Rates for patients >40 years of age were grouped and averaged because patient numbers were small after this time. A normally distributed frailty term for study was incorporated into the model on the log-hazard scale to account for potential between-study variability.^14^ The frailty term allows the precise estimates of mortality within each study to differ by introducing unobserved variation between studies. It is included because the studies cover a range of global populations with follow-up over different calendar periods. The choice of parametric model enables smoother results with interval-specific mortality, which are directly applicable to health economic analysis. Proportional hazards were assumed in secondary analysis between birth cohorts; the assumption was assessed with log-log plots of survival in each cohort against log time. Stata 16 was used for statistical analysis.

### Data Availability

The data used in this review are reconstructed IPD and thus do not directly correspond to real patients. The full reconstructed dataset is available on request.

### Standard Protocol Approvals, Registrations, and Patient Consents

No protocol or ethics approval was required for this work because the data are already anonymized and in the public domain.

## Results

### Systematic Review

**Figure 1:**
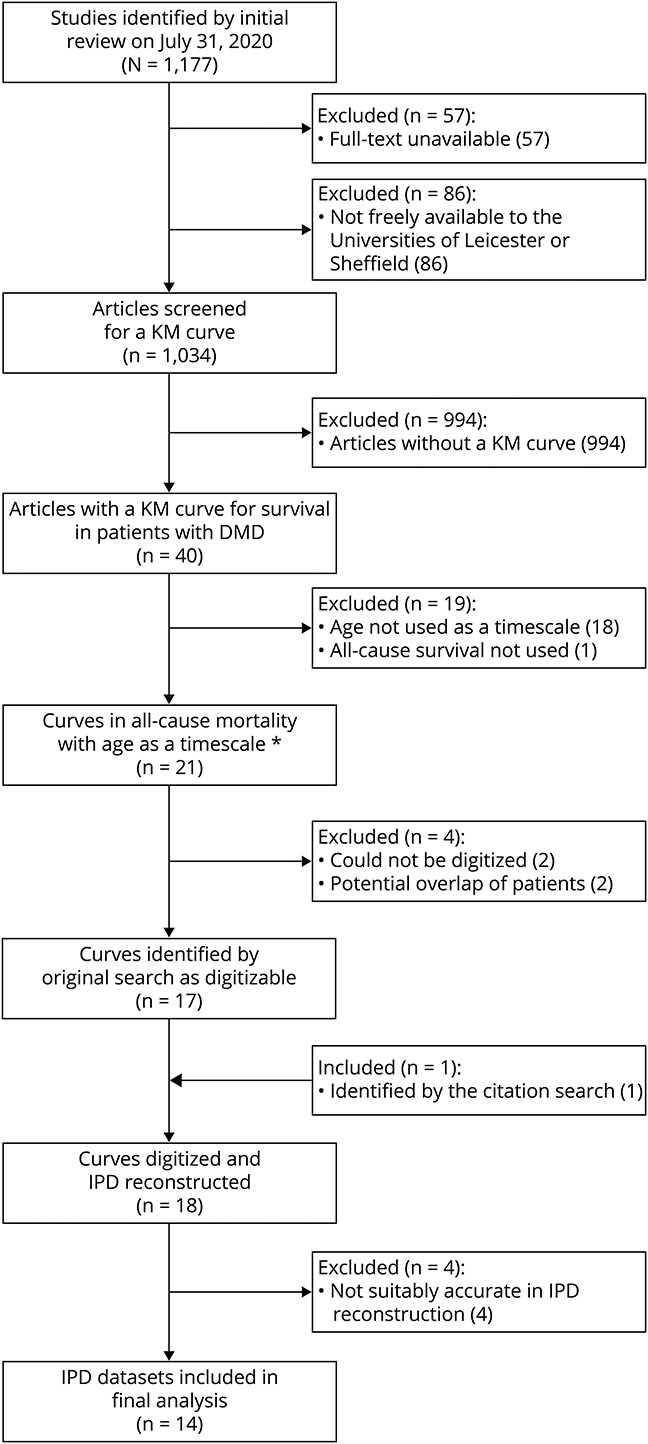
Flow of Identified DMD Mortality Articles on PubMed DMD = Duchenne muscular dystrophy; IPD = individual patient data; KM = Kaplan-Meier. *The KM curve in 1 article did not use age as a timescale, but the age of patients could be calculated.

The flowchart of the systematic review is shown in Figure 1. Of 1,177 results from the initial search described in Systematic Review in Methods, 21 articles contained at least 1 appropriate KM curve. Two were excluded for not being able to be digitized. Two more were excluded for a potential overlap of patients; in both instances, a larger study was conducted by the same authors in the same location that was eligible for inclusion, so the smaller study was excluded. Thus, 17 articles remained eligible for digitization. After the supplementary review of references in systematic reviews of DMD mortality, a further article was identified as appropriate for inclusion from the Landfeldt et al.^8^ review. This brought the final total number of articles to 18.

Eighteen articles were eligible for digitizing,^1-7,15-25^ contributing 3,131 total patients and 1,250 total deaths. Table 1 contains the key details of these articles. The studies were performed worldwide, including Europe, the United States, Chile, and Japan, and covered a range of birth, clinic admittance, and death cohorts, with the earliest being born in 1954 and the latest in the late 2000s. Table 2 details how studies were assigned to each birth cohort.

**Table 1:**
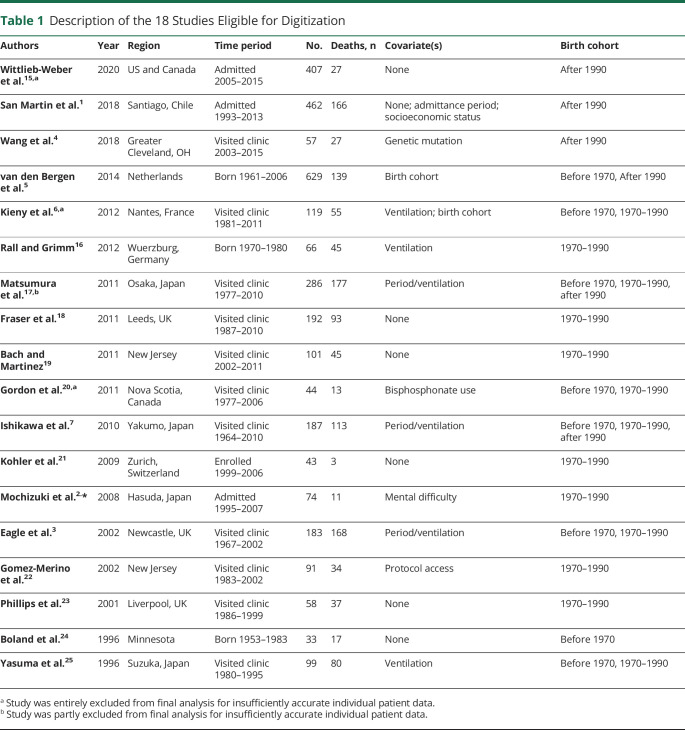
Description of the 18 Studies Eligible for Digitization

**Table 2:**
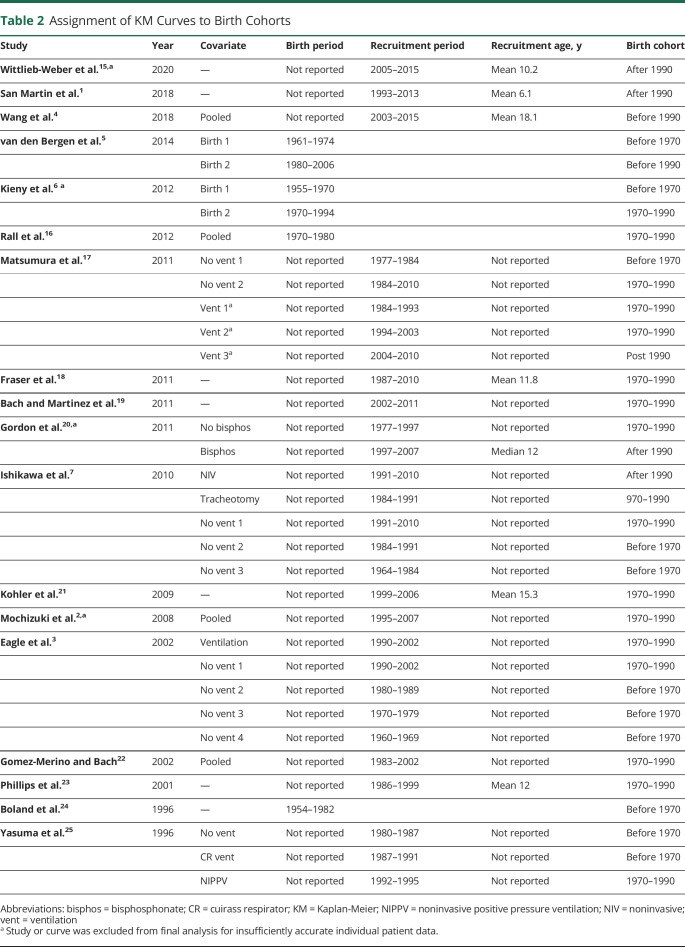
Assignment of KM Curves to Birth Cohorts

The results of the JBI tool are displayed in Table 3. Overall, articles were of fairly high quality; the most common issue was with exclusion criteria (criteria C5). Two articles performed slightly more poorly than the rest, 1 due to poor reporting of methods of diagnosis and analysis^19^ and the other to the article being an editorial and providing insufficient detail.^25^

**Table 3:**
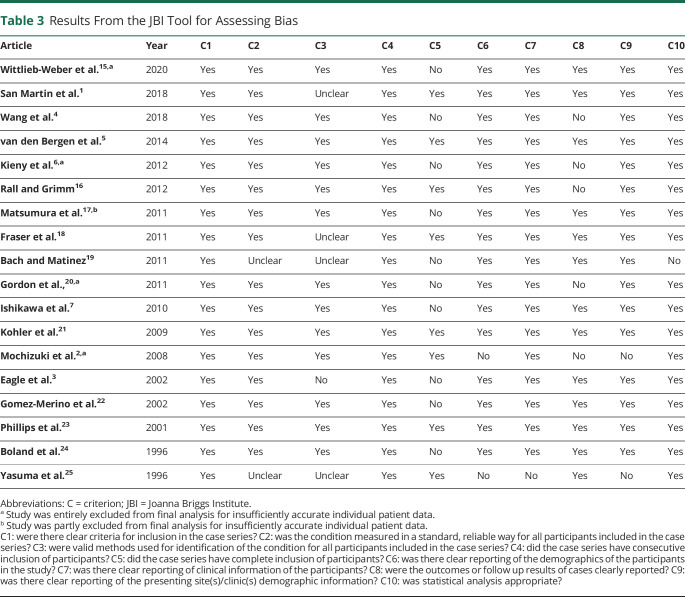
Results From the JBI Tool for Assessing Bias

### IPD Extraction

Of the KM curves from the 18 articles, 4 were not adequately reproduced by either of the 2 authors.^2,6,15,20^ The reason was that the number of deaths in each level of the curve was not reported, so the algorithm was much less accurate in data reconstruction. These studies were excluded from the analysis. In addition, 1 article contained only information on the number of deaths for some of the covariate levels^17^; IPD from these curves were included, but not from the other curves in this articles because deaths were again overestimated by both authors.

The digitized data from the remaining 14 studies yielded 2,283 patients and 1,050 deaths (compared to 1,049 in the original; 1 article produced 1 more digitized death than was reported^16^). The digitized data gave KM curves broadly very similar to those presented in the publications. The algorithm is least accurate when reproducing ages at death at the end of follow-up, so KM curve replication was poorest in the tails of some studies.^1,5,22^ In these studies, a small percentage of deaths were observed at late follow-up. Median survival was also compared between original and reproduced curves and was consistently reproduced and is presented in Table 4.

**Table 4:**
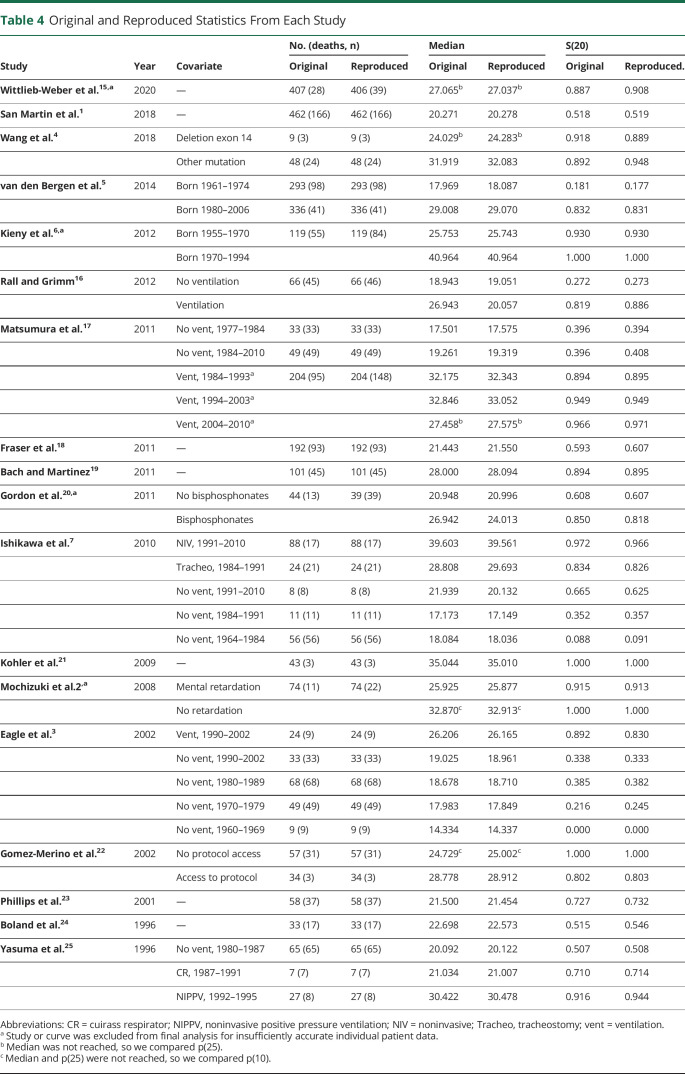
Original and Reproduced Statistics From Each Study

### Pooled Data Analysis

**Figure 2:**
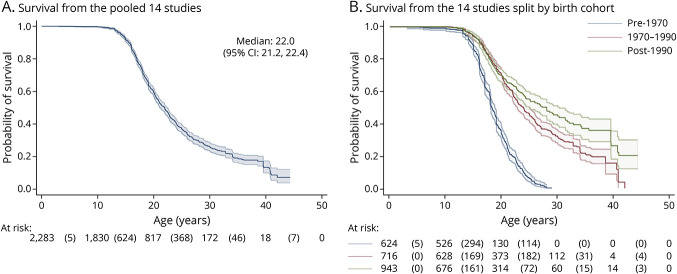
Kaplan-Meier Estimates, (A) Pooled and (B) Split by Birth Cohort, From the Digitized Data

The total follow-up time was 40,274 patient-years with a maximum age of 44.4 years. Median survival age, calculated with the KM estimator, was 22.0 years (95% CI 21.2, 22.4). Survival probabilities at 10, 20, 30, and 40 years were 99.8% (95% CI 99.4%, 99.9%), 59.5% (95% CI 56.9%, 61.9%), 26.1% (95% CI 23.5%, 28.8%), and 13.3% (95% CI 9.8%, 17.3%), respectively. Figure 2A illustrates the survival probabilities from the digitized data. The at-risk table shows the number of patients at risk at the beginning of the interval, and the number in parentheses is the number of deaths that occurred within the interval.

Secondary analysis suggested an improvement in life expectancy over time. The median survival age from the pre-1970 birth cohort was 18.3 years (95% CI 18.0, 18.9) compared to 24.0 years in the 1970 to 1990 birth cohort (95% CI 22.8, 25.0) and 28.1 years in the post-1990 birth cohort (95% CI 25.1, 30.3). Survival split by birth cohort is presented in Figure 2B. Log-log plots of survival in each cohort were approximately parallel, indicating that proportional hazards was an appropriate assumption.

The 5-year mortality rates per 1,000 person-years with CIs from the piecewise exponential model are given in Table 5, adjusted for between-study variability by including a shared frailty term. Mortality is averaged over patients >40 years of age because data are sparse.

**Table 5:**
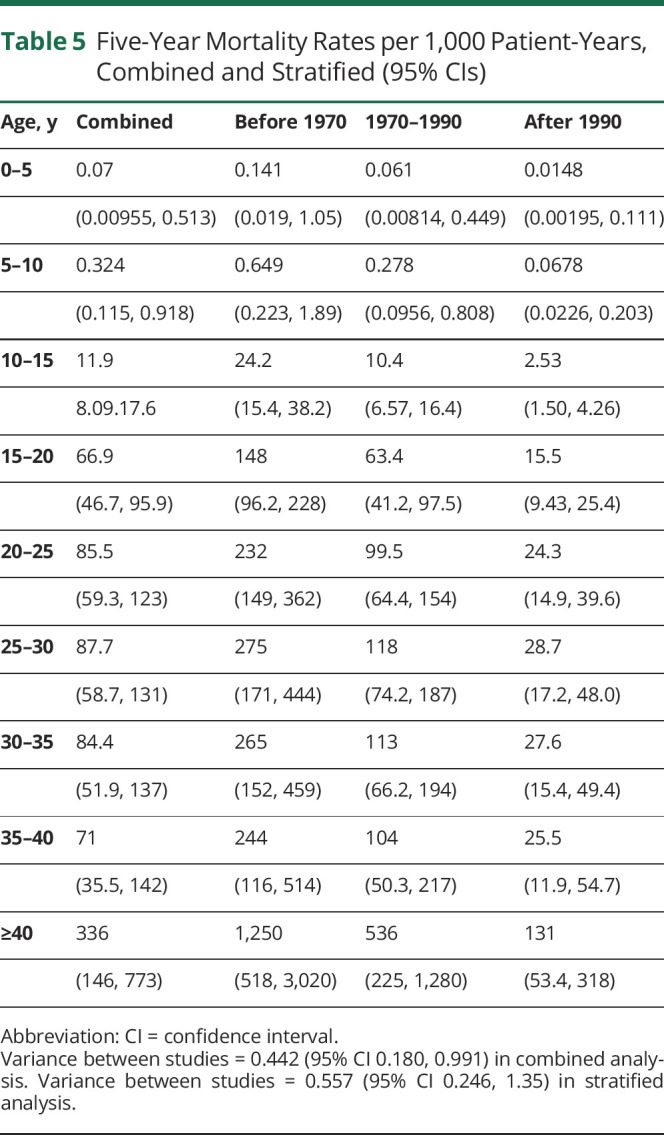
Five-Year Mortality Rates per 1,000 Patient-Years, Combined and Stratified (95% CIs)

Mortality is very low in patients with DMD between 0 and 10 years of age and increases with age. In the combined analysis, it was estimated that for every 1,000 patients 20 to 25 years of age, 86 would die each year, increasing to 336 each year for those >40 years of age. Uncertainty increases as patients age; more patients die or are censored, so there are fewer patients from which to estimate mortality rates. Mortality rates were much higher in the birth cohort from before 1970 compared to the later cohorts; 265 of every 1,000 patients with DMD 25 to 30 years of age who were born before 1970 died every year compared to just 27.6 a year for every 1,000 born after 1990.

As a sensitivity analysis, the studies that performed more poorly in the JBI tool^19,25^ were removed, and the nonparametric analysis was rerun. This yielded an overall median survival of 21.4 years (95% CI 20.8, 22.2) and median survival estimates of 18.1 years (95% CI 17.8, 18.7), 22.9 years (95% CI 22.0, 24.0), and 28.1 years (95% CI 25.1, 30.3) for patients born before 1970, between 1970 and 1990, and after 1990, respectively. These estimates are very similar to the original analysis.

## Discussion

Our work has provided a set of accessible age-specific all-cause mortality rates that can be incorporated into the natural history modeling of DMD. This is particularly important for economic decision modeling evaluating future health technologies/treatments not just in DMD but also as a framework for other rare diseases.^26^ Natural history models rely on reliable estimates of mortality throughout the disease pathway, and while the analysis is not without limitation, the only previous meta-analytic work of this nature provides a median estimate of survival,^8^ which, while useful, is not sufficiently granular for natural history modeling.

Early mortality is negligible both in the overall dataset and in each birth cohort. Median survival in the overall dataset was 22.0 years (95% CI 21.2, 22.4), but survival rates have increased over time, with a median survival of 28.1 years (95% CI 25.1, 30.3) in patients born after 1990. These results are consistent with other recent work.^8^ Moreover, these estimates may slightly underestimate median survival in more recently diagnosed patients because some of the articles that were excluded from the final analysis at the data reconstruction phase had KM curves of patients who either had not reached median survival by 30 years of age^15^ or had median estimates >30 years of age.^6,17^

Our work is comparable to the Landfeldt et al.^8^ review. The search terms were almost identical, and all articles included in their study were identified by our search. However, 6 of our 18 articles identified by the systematic review were not included in their review, which could be due to slightly differing search terms^2^ or differing exclusion criteria of DMD diagnosis.^18^ While our article relied only on a clinical, rather than genetic, diagnosis, the 2 studies produced very similar results, as did a subanalysis of our cohort excluding studies that were not included in the Landfeldt et al.^8^ review. Median survival was comparable between the reviews, with both identifying a marked improvement in life expectancy over time. However, our work also provides age-specific estimates of survival and mortality rates over the whole trajectory of a patient with DMD, allowing natural history models to incorporate mortality representative of the global population of patients with DMD.

This study highlights the importance of making the most of available data, an issue particularly pertinent to rare diseases such as DMD. Appropriate estimates of life expectancies are also important for other reasons; for instance, a better understanding of life expectancies and estimates of age-specific mortality for patients with DMD could help with disease management and planning of service provision, as well as counseling for parents and caregivers.

However, there are limitations with our work. First, access to the full IPD in the studies would be most desirable to avoid unnecessary study exclusion caused by a lack of knowledge of strata sample sizes and to eliminate any errors caused by data extraction. This study does provide generalizable methodology for situations when IPD are unavailable. We were also limited by restricted literature access and could not review 86 of the 1,177 identified articles. In addition, the assignment of patients to birth cohorts was not always straightforward, and knowledge of the exact period of birth of patients in each study would have been preferable.

A further complication from the lack of IPD is that all patients are assumed to have been followed up from birth. This introduces the possibility of immortal time bias in studies because it is an unintentional condition to survive beyond a certain point to be included in these studies. In other words, the sickest patients who die before being recruited to a study are ignored, so the population analyzed may be slightly healthier than the true underlying DMD population. The impact of this is probably minimal because studies that did report average ages at recruitment generally reported fairly young (<10 years of age) recruitment ages, but it is difficult to assess what impact, if any, this possible bias may have on the final results.

Further work could be undertaken investigating the impact of other covariates such as geographic location (which could affect standard of care), information on steroid treatment (such as type and duration of treatment, age at initiation), and ventilator use. Although these are interesting and pertinent questions, this would require a high level of harmonization between studies. Our birth cohort assignment should stratify between patient populations before and after steroid use became mainstay (after the publication of a number of trials in the late 1980s and early 1990s reporting beneficial effects of corticosteroid therapy^27^), but this obviously does not guarantee a clear comparison of steroid and nonsteroid users. The study populations vary globally and ethnically, with studies from North America, South America, Europe, and Asia. While an in-depth subanalysis by region would be desirable, it is a reflection of the rarity of the disease that these sources must be pooled together to obtain more precise estimates of survival rates and probabilities.

Extensions of the work could be to supplement and compare with results from the Cooperative International Neuromuscular Research Group. This group has conducted an international natural history study on 440 patients.^28^ This study included prospectively collected mortality data,^29^ but to date, no mortality analysis has been published. Comparison with this study would enable validation of these results in a US population because only 5 of the 14 included studies contained patients from the United States. Similar work could be completed in the United Kingdom by linking to large-scale population-linked electronic health record data, for example, the Clinical Practice Research Datalink. This would have the additional advantage of minimizing selection effects due to cohorts often being established at specialist centers.

We make several recommendations on the basis of our findings. This work highlights improvements in survival for patients with DMD over time; standards of care have improved. We emphasize the need for mortality collection to be considered in the design stage, especially in natural history/registry studies. We strongly advocate the need to include mortality data in any natural history mode, to accurately represent the whole natural history, regardless of whether IPD are available. When they are not available, we recommend using the DMD mortality statistics provided here or obtaining them through other means (such as a review of relevant records or similar systematic review of mortality rates).

## Glossary

CI: confidence interval
DMD: Duchenne muscular dystrophy
IPD: individual patient data
JBI: Joanna Briggs Institute
KM: Kaplan-Meier

## References

[1] San Martin PP, Solis FF, Cavada CG. Survival of patients with Duchenne muscular dystrophy. Revista Chilena de Pediatria. 2018;89(4):477. 30571821 10.4067/S0370-41062018005000704

[2] Mochizuki H, Miyatake S, Suzuki M, et al. Mental retardation and lifetime events of Duchenne muscular dystrophy in Japan. Intern Med. 2008;47(13):1207-1210. 18591841 10.2169/internalmedicine.47.0907

[3] Eagle M, Baudouin SV, Chandler C, Giddings DR, Bullock R, Bushby K. Survival in Duchenne muscular dystrophy: improvements in life expectancy since 1967 and the impact of home nocturnal ventilation. Neuromuscul Disord. 2002;12(10):926-929. 12467747 10.1016/s0960-8966(02)00140-2

[4] Wang M, Birnkrant DJ, Super DM, Jacobs IB, Bahler RC. Progressive left ventricular dysfunction and long-term outcomes in patients with Duchenne muscular dystrophy receiving cardiopulmonary therapies. Open Heart. 2018;5(1):e000783. 29531771 10.1136/openhrt-2018-000783 PMC5845428

[5] Bergen JC, Ginjaar HB, Essen AJ, et al. Forty-five years of Duchenne muscular dystrophy in the Netherlands. J Neuromuscul Dis. 2014;1(1):99-109. 27858664

[6] Kieny P, Chollet S, Delalande P, et al. Evolution of life expectancy of patients with Duchenne muscular dystrophy at AFM Yolaine de Kepper centre between 1981 and 2011. Ann Phys Rehabil Med. 2012;56:443-454. 10.1016/j.rehab.2013.06.002 23876223

[7] Ishikawa Y, Miura T, Ishikawa Y, et al. Duchenne muscular dystrophy: survival by cardiorespiratory interventions. Neuromuscul Disord. 2010;21(1):47-51. 21144751 10.1016/j.nmd.2010.09.006

[8] Landfeldt E, Thompson R, Sejersen T, McMillan HJ, Kirschner J, Lochmuller H. Life expectancy at birth in Duchenne muscular dystrophy: a systematic review and meta-analysis. Eur J Epidemiol. 2020;35(7):643-653. 32107739 10.1007/s10654-020-00613-8 PMC7387367

[9] Moher D, Liberati A, Tetzla J, Altman DG; PRISMA Group. Preferred Reporting Items for Systematic Reviews and Meta-Analyses: the PRISMA statement. PLoS Med. 2009;6(7):e1000097. 19621072 10.1371/journal.pmed.1000097 PMC2707599

[10] Moola S, Munn Z, Tufanaru C, et al. Chapter 7: Systematic Reviews of Etiology and Risk: Joanna Briggs Institute Reviewer's Manual. Joanna Briggs Institute; 2017. Accessed November 13, 2020. reviewersmanual.joannabriggs.org/.

[11] Rohatgi A. WebPlotDigitizer. 2020. Accessed November 13, 2020. automeris.io/WebPlotDigitizer/.

[12] Guyot P, Ades AE, Ouwens MJNM, Welton NJ. Enhanced secondary analysis of survival data: reconstructing the data from published Kaplan-Meier survival curves. BMC Med Res Methodol. 2012;12(1):9. 22297116 10.1186/1471-2288-12-9 PMC3313891

[13] Wei Y, Royston P. Reconstructing time-to-event data from published Kaplan-Meier curves. Stata J. 2017;17(4):786-802. 29398980 PMC5796634

[14] Crowther MJ, Riley RD, Staessen JA, Wang J, Gueyffier F, Lambert PC. Individual patient data meta-analysis of survival data using Poisson regression models. BMC Med Res Methodol. 2012;12:34. 22443286 10.1186/1471-2288-12-34 PMC3398853

[15] Wittlieb-Weber CA, Knecht KR, Villa CR, et al. Risk factors for cardiac and non-cardiac causes of death in males with Duchenne muscular dystrophy. Pediatr Cardiol. 2020;41(4):764-771. 32016582 10.1007/s00246-020-02309-y PMC7328368

[16] Rall S, Grimm T. Survival in Duchenne muscular dystrophy. Acta Myologica. 2012;31(2):117-120. 23097602 PMC3476855

[17] Matsumura T, Saito T, Fujimura H, Shinno S, Sakoda S. A longitudinal cause-of-death analysis of patients with Duchenne muscular dystrophy. Rinsho Shinkeigaku. 2011;51(10):743-750. 22019865 10.5692/clinicalneurol.51.743

[18] Fraser LK, Childs A, Miller M, et al. A cohort study of children and young people with progressive neuromuscular disorders: clinical and demographic profiles and changing patterns of referral for palliative care. Palliat Med. 2011;26(7):924-929. 21908523 10.1177/0269216311419989

[19] Bach JR, Martinez D. Duchenne muscular dystrophy: continuous noninvasive ventilatory support prolongs survival. Respir Care. 2011;56(6):744-750. 21333078 10.4187/respcare.00831

[20] Gordon KE, Dooley JM, Sheppard KM, MacSween J, Esser MJ. Impact of bisphosphonates on survival for patients with Duchenne muscular dystrophy. Pediatrics. 2011;127(2):e353-e358. 21242224 10.1542/peds.2010-1666

[21] Kohler M, Clarenbach CF, Bahler C, Brack T, Russi EW, Bloch KE. Disability and survival in Duchenne muscular dystrophy. J Neurol Neurosurg Psychiatry. 2009;80(3):320-325. 18713792 10.1136/jnnp.2007.141721

[22] Gomez-Merino E, Bach J. Duchenne muscular dystrophy. Am J Phys Med Rehabil. 2002;81(6):411-415. 12023596 10.1097/00002060-200206000-00003

[23] Phillips MF, Quinlivan RCM, Edwards RHT, Calverley PMA. Changes in spirometry over time as a prognostic marker in patients with Duchenne muscular dystrophy. Am J Respir Crit Care Med. 2001;164(12):2191-2194. 11751186 10.1164/ajrccm.164.12.2103052

[24] Boland BJ, Silbert PL, Groover RV, Wollan PC, Silverstein MD. Skeletal, cardiac, and smooth muscle failure in Duchenne muscular dystrophy. Pediatr Neurol. 1996;14(1):7-12. 8652023 10.1016/0887-8994(95)00251-0

[25] Yasuma F, Sakai M, Matsuoka Y. Effects of noninvasive ventilation on survival in patients with Duchenne's muscular dystrophy. Chest. 1996;109(2):590. 8620758 10.1378/chest.109.2.590

[26] Hatswell AJ, Chandler F. Sharing is caring: the case for company-level collaboration in pharmacoeconomic modelling. Pharmacoeconomics. 2017;35(8):755-757. 28528523 10.1007/s40273-017-0516-2

[27] Moxley RT III, Pandya S, Ciafaloni E, Fox DJ, Campbell K. Change in natural history of Duchenne muscular dystrophy with long-term corticosteroid treatment: implications for management. J Child Neurol. 2010;25(9):1116-1129. 20581335 10.1177/0883073810371004

[28] Longitudinal Study of the Relationship Between Impairment, Activity Limitation, Participation and Quality of Life in Persons With Confirmed Duchenne Muscular Dystrophy (DMD). 2005. Accessed April 2, 2020. clinicaltrials.gov/ct/show/NCT00468832?order=1.

[29] McDonald CM, Henricson EK, Abresch RT, et al. Long-term effects of glucocorticoids on function, quality of life, and survival in patients with Duchenne muscular dystrophy: a prospective cohort study. Lancet. 2018;391(10119):451-461. 29174484 10.1016/S0140-6736(17)32160-8

